# Optimization of a Multi-Residue Analytical Method during Determination of Pesticides in Meat Products by GC-MS/MS

**DOI:** 10.3390/foods11192930

**Published:** 2022-09-20

**Authors:** Sang-Hyeob Lee, Se-Yeon Kwak, Aniruddha Sarker, Joon-Kwan Moon, Jang-Eok Kim

**Affiliations:** 1 School of Applied Biosciences, Kyungpook National University, Daegu 41566, Korea; 2 School of Plant Resources and Landscape Architecture, Hankyong National University, Anseong 17579, Korea

**Keywords:** pesticides determination, SPE cartridges, GC-MS/MS, multi-residue analysis, meat products, matrix effect

## Abstract

In this study, a multi-residue analysis was developed for 32 compounds, including pesticides and metabolites, in five meat products using gas chromatography-tandem mass spectrometry (GC-MS/MS). The validation of the developed analytical method was also evaluated in accordance with Codex Alimentarius guidelines. Aminopropyl (NH_2_), C_18_, and florisil solid phase extraction (SPE) cartridges were used to evaluate and optimize the cleanup procedure of the tested samples prior to GC-MS/MS analysis. Based on the analytical performance, the C_18_ SPE cartridge was deemed to be the most suitable among the examined SPE cartridges. The optimized method demonstrated that 29 out of 32 tested compounds acquired good linearity (R^2^ ≥ 0.99), and 25 tested compounds displayed the method limit of quantification (MLOQ) ≤ 0.01 mg/kg. Out of the 32 tested compounds, only 21 compounds met the acceptable analytical criteria for the lard and tallow samples, compared to 27 compounds in the beef, pork, and chicken samples that falls within the acceptable standards for recovery (70–120%) and analytical precision (relative standard deviation RSD ≤ 20%). The average matrix effect was widely varied (20.1–64.8%) in the studied meat samples that were affected by either ion enhancement or suppression. In particular, in the lard sample, 13 compounds showed poor recovery and analytical precision due to ion suppression. Thus, the matrix effect (ME) was considered a critical factor during multi-residue pesticide analysis in different meat products. In conclusion, this developed analytical method can be used as a routine monitoring system for residual pesticide analysis in livestock products with acceptable analytical standards. Further meticulous analytical studies should be optimized and validated for multi-residue pesticide analysis in diversified meat products.

## 1. Introduction

The widespread consumption of livestock products, along with the improvement of the living standards, has placed a greater emphasis on the quality and safety of livestock food products [1]. The raw meat generation, processing, distribution, and marketing were considered key entry points for contaminants including pesticides and metabolites, which are the primary direct risk factors affecting the safe production and consumption of livestock food products [2,3]. Pesticides are introduced to livestock animals to prevent infestations, but they may persist in livestock food products such as edible meat, milk, and eggs. Eventually, the accumulation pesticides in the human body through the trophic transfer of contaminated foods derived from livestock has been shown to disrupt reproductive function, cause deformities, promote acute or chronic toxicity, and affect the endocrine system of the human body [4]. To address these concerns, a strong emphasis has been placed on the food hazard assessment of residual pesticides in livestock food products. Thus, to standardize the maximum residue limits (MRLs) in livestock products, it is essential to optimize and establish a reliable multi-residue analytical method that can be applied to the routine monitoring of residual pesticides in livestock meat products by regulatory agencies [5,6].

While multi-residue analytical methods have a higher analysis efficiency, their precision and reliability may be slightly lower than those of individual-residue analytical methods [7]. Thus, multi-residue analytical methods are developed with a focus on enhancing their analysis efficiency on the basis of appropriate international standards. The developed multi-residue methods are particularly suitable for residual pesticide detection, which involves the quick, easy, cheap, effective, rugged, and safe (QuEChERS) extraction and the use of liquid chromatography (LC), gas chromatography (GC) and tandem mass spectrometry [8]. Concisely, QuEChERS extraction involves two processes of extraction, making it a simple pretreatment method. It also allows the extraction and purification of various pesticide compounds. Although its purification efficiency is typically low, QuEChERS extraction is currently the most commonly used method when combined with mass spectrometry, which ensures high selectivity and sensitivity [9]. The use of QuEChERS extraction for the analysis of animal food products, however, may result in challenges related to analytical device maintenance and reliability of both quantitative and qualitative results, due to its intrinsic low purifying efficiency as previously mentioned [10,11].

Compared to agricultural products, livestock food products have a lower water content. However, their fat, lipid, and protein contents are high, which necessitates an effective purification process for their removal [2,3]. As evaluated through GC-MS/MS, in particular, the ME strengthens with an increase in the fat or lipid content of samples [12]. The column lifetime may also shorten upon the continuous injection of livestock food product samples [13]. Moreover, changes in the peaks associated with pesticide compounds could result in inaccurate quantification analysis [11]. The effective removal of fats and phospholipids can be achieved through the use of a SPE cartridge or a freeze-out technique and via acetonitrile/hexane partitioning. The Pesticide Analytical Manual (PAM) developed by the US Food and Drug Administration (FDA) [14] contains the FDA-recommended purification methods involving gel permeation chromatography, petroleum ether/acetonitrile partitioning, and cleanup using florisil for the removal of fats from samples [15]. However, such methods require expensive special equipment for pretreatment, and the pretreatment methods exhibit substantially low efficiency and high maintenance costs [16]. Moreover, the optimization of the analytical conditions of such methods for the accurate fractionation between fat and pesticide compounds is difficult, making them unsuitable for general analysis. It is thus necessary to establish a validated pretreatment method and to develop a device-specific analytical method with outstanding reproducibility and recovery through the optimization of the solvent partition used in other analytical methods.

Although previous studies have reported the validation of analytical approaches for pesticide determination in fish and livestock products [3], there are still several uncertainties regarding the analytical standards during determination of pesticides in commercial meat products due to varying fat contents and compositions. Considering these limitations, the meat products used in this study were beef (ruminant), pork (nonruminant), and chicken (poultry). In addition, because some target pesticides are oil soluble, tallow and lard were used.

Considering the current research trend and limitations, specific objectives of this study are to: (i) develop a multi-residue analysis method to detect 32 compounds (29 pesticides and three metabolites) in five meat products purchased from the domestic market in Korea; (ii) optimize the instrumental conditions and clean-up process using three different SPE cartridges, which can be easily applicable during a reliable monitoring system; and (iii) validate the developed analytical method according to Codex Alimentarius guidelines [17].

## 2. Materials and Methods

### 2.1. Chemicals and Reagents

A total of 29 types of pesticides were selected based on the abundance of reported pesticides in livestock products by previous studies [2,3], including esfenvalerate, an insecticide; 19 fungicides; 7 herbicides, a plant growth regulator (PGR); chinomethionate, used as a fungicide and insecticide; and chlorpropham, used as an herbicide and PGR. The detailed information of the studied pesticides including their physicochemical properties and the Codex Alimentarius MRLs on livestock products in Korea are available in the supporting file (Table S1). The 29 pesticides included both polar and nonpolar compounds with log P_ow_ ranging between −0.17 and 6.24. Most of the pesticides used (19 out of 29) were nonpolar compounds with log P_ow_ ≥ 3. The standards for each pesticide were purchased from AccuStandard Inc. (New Haven, CT, USA). Based on the solubility of each pesticide, a stock solution of 100–500 mg/L was prepared using acetone, acetonitrile, and methanol as solvents, whereas the mixed standard solution for the pesticides was prepared by dilution of the pesticides in acetone to set a final concentration of 5.0 mg/L for each compound.

Acetone, acetonitrile, and n-hexane (Wako Pure Chemical Industries, Ltd., Osaka, Japan) were used in the sample pretreatment before analytical studies, whereas methanol (MeOH) and dichloromethane (DCM) (Merck, Darmstadt, Germany) were used for high-performance liquid chromatography (HPLC) analysis. The roQ™ QuEChERS Extraction Packets (6 g magnesium sulfate and 1.5 g sodium acetate) buffer salts from Phenomenex Inc. (Torrance, CA, USA) were used in the sample extraction, and Sep-Pak C_18_ SPE cartridges (1 g, 6 cc) from Waters Corporation (Milford, MA, USA) were used in purification.

### 2.2. Sample Selection

Following optimization regulatory guidelines [18], this study optimized an analytical method for the determination of specific pesticides in various commercial meat products in Korea. As presented in Table S2, meat is divided into red muscle, white muscle, and offal. For red muscle, beef and pork were used, and for white muscle, chicken was used. In addition, extracted fats such as tallow and lard were used, as certain pesticides are oil- soluble and could accumulate in the fats of livestock products [19]. Critical selection criteria were chosen while the meat was cut, that is, the sirloin, belly, and drumstick were separated and used for beef, pork, and chicken, respectively. This is due to the fact that these were the meat parts that are highly consumed, have a high fat content and the fat extracted during analysis is a well-known coextractive that may cause considerable ME in GC-MS/MS analysis [20]. The aforementioned parts were thus selected as it was predicted that sample preparation could be time consuming, due to the need to eliminate as much extracted fat as possible in order to develop an optimal analytical method. The purchased beef, pork, chicken, tallow and lard were ground in a blender with dry ice for a set period of time until the unified powder was formed, and the resulting powder forms were placed in polyethylene bags for storage at −20 °C in a freezer prior to analysis.

### 2.3. GC-MS/MS Analytical Conditions

For the GC-MS/MS analysis of studied pesticides in livestock products, GCMS-TQ8050 (Shimadzu, Kyoto, Japan) was used. The columns used in the analysis were ZB-5MS Plus (30 m × 0.25 mm, 0.25 µm, Phenomenex Inc., Torrance, CA, USA). The carrier gas was helium at a flow rate of 1.5 mL/min, and the Q2 collision gas was argon. The interface temperature was set at 300 °C for the splitless injection of 2 µL. The oven temperature was initially set at 90 °C, which was maintained for 3 min; it was then increased to 120 °C at a rate of 20 °C/min; and then increased at a rate of 8 °C/min to 300 °C and maintained for 3 min. The GC-MS/MS analytical conditions are presented in detail in Table A2.

### 2.4. Sample Preparation

As shown in Figure A2, 5 g of the homogenized samples was accurately weighed and placed in a 50 mL centrifuge tube. After the addition of 15 mL of acetonitrile containing 1% acetic acid, the mixture was vortexed for 10 min for extraction. Next, 6 g of magnesium sulfate and 1.5 g of sodium acetate were added. The mixture was vortexed and centrifuged at 4000 rpm for 10 min.

To compare the solid phase extraction method, the adsorbent used in the dSPE was 100 mg PSA and 100 mg C_18_ following the modified QuEChERS method [21]. The 1.0 mL supernatant of the centrifuged extract was transferred to a microtube with 100 mg PSA and 100 mg C18. The microtubes were vortexed for 1 min with a vortexer. Then, the tubes were centrifuged at 15,000 rpm for 5 min. The organic solvent layer (upper layer) was filtered through a high-quality polyvinylidene fluoride (PVDF, 0.2 µm) membrane filter into an autosampler vial for GC-MS/MS analysis.

In the optimized SPE method, the supernatant of the centrifuged extract (4 mL) was taken to be dried using nitrogen at 40 °C and then redissolved in 4 mL DCM/MeOH (99/1, *v*/*v*). The redissolved solution was used as the loading solution in SPE purification. Furthermore, 6 mL DCM was injected into the 1 g C_18_ SPE cartridge to be purged out; then, 4 mL of the loading solution was injected. Next, with 3 mL DCM/MeOH (99/1, *v*/*v*), a total of 7 mL of the eluent solution, was evaporated to dryness under nitrogen flow at 40 °C, and the sample was redissolved in 2 mL acetone and then filtered through a PVDF membrane filter (0.2 µm) prior to be used for GC-MS/MS analysis.

### 2.5. Method Validation

The multi-residue analytical method for the five meat products was validated based on selectivity, limit of detection (LOD), limit of quantification (LOQ), linearity, accuracy, and precision (repeatability-intraday, reproducibility-interday), according to the Codex Alimentarius guidelines (CAC/GL 40) [3].

To evaluate the selectivity of the analytical method, blank samples and recovery samples with the standard solutions were compared based on the absence of the interfering peak in the blank sample. For LOD and LOQ, the signal-to-noise (S/N) ratio corresponding to the chromatogram was set to 3 and 10, respectively. After confirming the analytical procedure, the method limit of quantification (MLOQ) was calculated using the amount of sample and the final volume, as shown by the following equation:

(1) $$\mathrm{MLOQ}\;\left(\mathrm{mg}/\mathrm{kg}\right)=\left[\frac{\mathrm{Minimum}\;\mathrm{detected}\;\mathrm{amount}\;\mathrm{in}\;\mathrm{the}\;\mathrm{instrument}\;\left(\mathrm{ng}\right)}{\mathrm{Injection}\;\mathrm{volume}\;\left(\mu L\right)}\;\right]\times \left[\frac{\mathrm{Volume}\;\mathrm{of}\;\mathrm{sample}\;\mathrm{solution}\;\left(\mathrm{mL}\right)}{\mathrm{Weight}\;\mathrm{of}\;\mathrm{sample}\;\left(g\right)}\right]$$

To evaluate the linearity of the analytical method in consideration of the ME, a matrix-matched calibration was established. The samples that were not treated with pesticides were diluted to contain 75% of the extraction solution, and were prepared following the same pretreatment method developed in this study. The resulting solution served as a standard solution. The concentration range used for the calibration was 0.002–0.1 mg/L. Calibration curves and correlation coefficient (*R*^2^) were calculated by the peak area for each matrix-matched solution.

To evaluate the accuracy (expressed as recovery) and precision (as relative standard solution, RSD) of the analytical method, a mixture of standard solution was added to each blank meat sample. The treatment was of four different spiked levels, and for the five representative meat products, each sample was analyzed for the concentration corresponding to MLOQ, 2 MLOQ, 10 MLOQ, and 50 MLOQ of this newly-proposed analytical method. The analysis was repeated five times, and the average recovery and RSD were calculated to assess the accuracy, repeatability, and reproducibility of the analytical method. In particular, inter-day precision, expressed as reproducibility, was evaluated over two different days.

In addition, as it is possible for the ME to arise in an analytical method based on mass spectrometry due to the extracted compounds from each sample, ME evaluation is necessary. Thus, to estimate the ME of pesticides in each sample, the linear regression slope from the matrix-matched calibration curve and the linear regression slope from the calibration curve of the standard solution without the matrix were used in the following equation [22]:

(2) $$\mathrm{ME}\;\left(\%\right)=\left[\frac{\left(\mathrm{Slope}\;\mathrm{of}\;\mathrm{matrix}\;\mathrm{matched}\;\mathrm{calibration}\;\mathrm{curve}\right)}{\left(\mathrm{Slope}\;\mathrm{of}\;\mathrm{standard}\;\mathrm{solution}\;\mathrm{calibration}\;\mathrm{curve}\right)}-1\right]\times 100$$

## 3. Results and Discussion

### 3.1. Optimization of Instrumental Conditions

To optimize the multiple reaction monitoring (MRM) conditions for the 29 parent compounds and 3 metabolites used in this study, a full scan and product ion scan were performed using a GC-MS/MS instrument. The mass range was set to 50–550 m/z, and a full scan was performed using 1 and 2 mg/kg standard solutions. Based on the full scan results, the compounds with high sensitivity and selectivity were selected as precursor ions having mass values of preferably ≥200 *m*/*z*. Using these selected precursor ions, a product ion scan was performed for varying collision-induced dissociation (CID) energy levels (3–42 eV). The product ions exhibiting the highest sensitivity were selected as quantification ions, whereas those exhibiting the second highest sensitivity were selected as qualification ions. Lastly, for the evaluation of the selectivity and reliability of the analytical method for each compound, one precursor and two product ions, as well as two precursor and one product ion were selected to satisfy at least the 3.0 required identification points. The resulting optimal MRM conditions are shown in Table A1.

**Figure 1:**
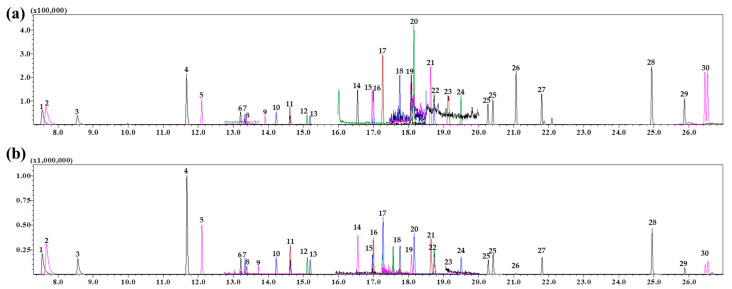
Comparison between split and splitless injections based on peak height and sensitivity for the different scale (10 folds) in GC-MS/MS. (**a**) Total ion chromatogram (ratio 10) of pesticides standard mixture (50 ng/mL), which was obtained using the split injection method; (**b**) Total ion chromatogram) of pesticides standard mixture (50 ng/mL), which was obtained using the splitless injection method. Peaks: 1: PAM, 2: dichlorobenil, 3: 3,5-dichloroaniline, 4: diphenylamine, 5: chlorpropham, 6: simazine, 7: dimethipin, 8: atrazine, 9: quintozene, 10: cyanazine, 11: pentachloroaniline, 12: vinclozoline, 13: alachlor, 14: phthalide, 15: pendimethalin, 16: cyprodinil, 17: fluopyram, 18: chinomethionat, 19: picoxystrobin, 20: flutriafol, 21: thifluzamide, 22: flusilazole, 23: fenoxanil, 24: penthipyrad, 25: propiconazole, 26: epoxiconazole, 27: fenamidone, 28: esfencalerate, 29: flumioxazine, 30: difenoconazole.

To determine the injection mode to be employed during GC-MS/MS measurements, split and splitless injections were compared. The results of a pilot study showed that the sensitivity of a mixture of standard solution was reduced through continuous injections of livestock samples in split injection mode. This deteriorated the reliability of qualitative data due to the changes in instrument LOD and LOQ upon repeated injections. As a result, the splitless injection method was selected to ensure a stable and high sensitivity upon repeated injections, as shown in Figure 1. Comparing the injection volumes of 1 and 2 μL into the GC-MS/MS instrument, the latter yielded more outstanding results; thus, 2 μL was set as the injection volume for the splitless injection mode. The optimized GC-MS/MS conditions in our study using the splitless mode was indicative of a sensitive and stable analytical performance, and could be helpful in other food commodities for multi-residual pesticides and set the regulation on MRLs.

### 3.2. Optimization of Sample Preparation

#### 3.2.1. Comparison of the Solid Phase Extraction Method

The samples of the studied meat products show a high matrix content including fats and proteins. The lipids, in particular, are easily dissolved in extraction solvents due to their high solubility in organic solvents, which should be purified by a suitable pretreatment prior to GC analysis [23,24]. Thus, the dispersive SPE (dSPE) and SPE cartridges that are most commonly used in the multi-residue analytical method for various pesticides were compared with respect to purification efficiency. The adsorbent used in the dSPE was 100 mg PSA and 100 mg C_18_ following the modified QuEChERS method [21], and for the SPE cartridge, 1 g C_18_ was used. The cleanup procedure of the beef sample using these adsorbents led to the TICs shown in Figure A1. Among the 32 target compounds, 17 compounds with log Pow > 3 were detected within a retention time (RT) of 14.00–19.63 min.

In this context, a higher ME causes more critical problems in quantification. Notably, fats and nonpolar compounds are factors that increase the ME. These compounds show a similar level of relative nonpolarity to the target compounds, and the RT in the column is also similar. Thus, adequate cleanup and separation are necessary for accurate quantification. In Figure A1a, an excited state on the baseline for a RT of 16.00–20.00 min can be seen on the chromatogram from the dSPE cleanup step. On the contrary, in Figure A1b, a ground state on the baseline for a RT of 16.00–20.00 min can be seen on the chromatogram from the C_18_ SPE cleanup step. The result indicated that the SPE cleanup step led to adequate adsorption of fats and nonpolar compounds in the matrix to produce a low ME and high purification efficiency. SPE was thus used in the analytical method designed for meat product samples, and the efficiency of removing co-extracts, including fats, was compared by varying the adsorbents in the SPE cartridge.

#### 3.2.2. Optimization of the SPE Method

**Figure 2:**
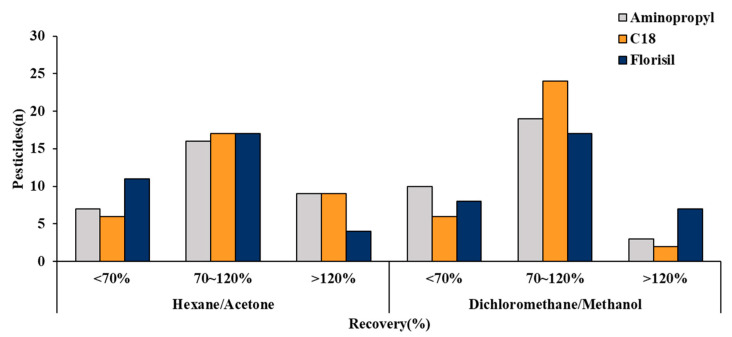
Comparison of recoveries for the 32 compounds obtained using three different SPE sorbents and two elution solvents.

The SPE method shows different purification efficiencies according to the type of adsorbent. Hence, three adsorbents, aminopropyl, C_18_, and florisil were compared. As the SPE cartridge also exhibits variations in the extracted amount of the target compound and matrix according to the extraction solvent, a suitable solvent should be selected. While the recovery of compounds is improved as the solvent extracting power increases, the amount of the extracted matrix also increases, thereby reducing the purification efficiency. Conversely, a reduction in the solvent extracting power could cause low recovery of pesticides, dissatisfying the recovery criteria [25]. Thus, the common extraction solvents for the SPE cartridge, which were hexane/acetone and DCM/MeOH combinations, were compared. The DCM/MeOH solvent was prepared in the ratios of 100/0, 99/1, and 98/2 *v*/*v*, which was dribbled into the cartridge in the increasing order of solvent extracting power. The hexane/acetone solvent has a lower extraction strength than the DCM/MeOH combination; thus, the hexane/acetone solvent was prepared with a higher proportion of acetone in the ratio of 95/5, 90/10, and 80/20 *v*/*v*. The solvent was injected into the cartridge and the resulting recovery was compared. As shown in Figure 2, the recovery pattern varied according to the SPE adsorbent and extraction solvent.

Overall, a higher proportion of compounds extracted using the C_18_ SPE cartridge satisfied the recovery criteria. Notably, the highest proportion of compounds (24 compounds) that satisfied the 70–120% recovery criteria were extracted using the DCM/MeOH solvent. Extraction using the hexane/acetone combination resulted in 16–17 compounds that satisfied the 70–120% recovery criteria, with little difference among the adsorbents. In contrast, the DCM/MeOH solvent demonstrated significant adsorbent variation. Thus, with a higher proportion of compounds satisfying the 70–120% recovery criteria, the DCM/MeOH combination was selected as the extracting solvent, and C_18_ was chosen as the adsorbent to optimize the analytical method.

Finally, the multi-residue analytical method for the determination of 32 compounds from the five meat products was developed and optimized, as discussed in Section 3.1 and Section 3.2. The details of the sample preparation are presented in Section 2.4 and Figure A2.

### 3.3. Selectivity, Linearity, and Limit of Quantification

To determine the selectivity of the analytical method toward the 29 target pesticides and three metabolites, the chromatograms of the standard solutions, blank samples, and recovery samples with standard solutions were compared. The application of the established method with the use of matrix-matched solutions yielded RT, and m/z values for all 32 compounds and interfering compounds were absent. These results suggest the high levels of separation and selectivity of the analytical method developed in this study.

As shown in Table 1, the MLOQ of the analytical method was 0.005 mg/kg for 26 compounds, 0.01 mg/kg for two compounds, and 0.02 mg/kg for one compound. Moreover, MLOQ was not calculated for captan, chlorothalonil, and dimethipin, as these three compounds were shown to be undetected due to the ME in the matrix-matched calibration. However, these compounds could be detected using only the standard solutions. In particular, captan and chlorothalonil are primarily known to display a tendency to decompose on GC columns during analysis of the agricultural products and livestock product samples [26,27,28].

In addition, to assess the linearity of the method to the target compounds and reflect the ME, the matrix-matched solutions were injected to the GC-MS/MS instrument at 0.002–0.1 mg/L in 2 μL volume for each concentration. Analysis results showed that all compounds, except the three undetected compounds, had *R*^2^ between 0.9850 and 0.9999, which satisfied the Codex Alimentarius criteria (*R*^2^ ≥ 0.99). Thus, the novel analytical method was verified to exhibit moderate linearity for quantification.

### 3.4. Accuracy and Precision

In Korea, due to the lack of MRLs in all types of meat products, including the 32 target compounds in this study, it is necessary to uniformly manage the pesticide level of the positive list system to below 0.01 mg/kg; therefore, the recovery test was performed. To evaluate the accuracy of the analytical method, the recovery test was repeated five times with four concentration levels, i.e., 10, 100, 500, and 5 µg/kg (the minimum MLOQ), for the five representative meat products. The criteria for accuracy and precision were based on the residue analytical method required by the Codex Alimentarius guidelines [3]. A suitable range of recovery for an analytical method for residual pesticides is as follows: 60–120% and below 32% RSD at 5 and 10 µg/kg, 70–120% and 22% RSD at 100 µg/kg, and 70–110% and 18% RSD at 500 µg/kg.

#### 3.4.1. Accuracy

**Figure 3:**
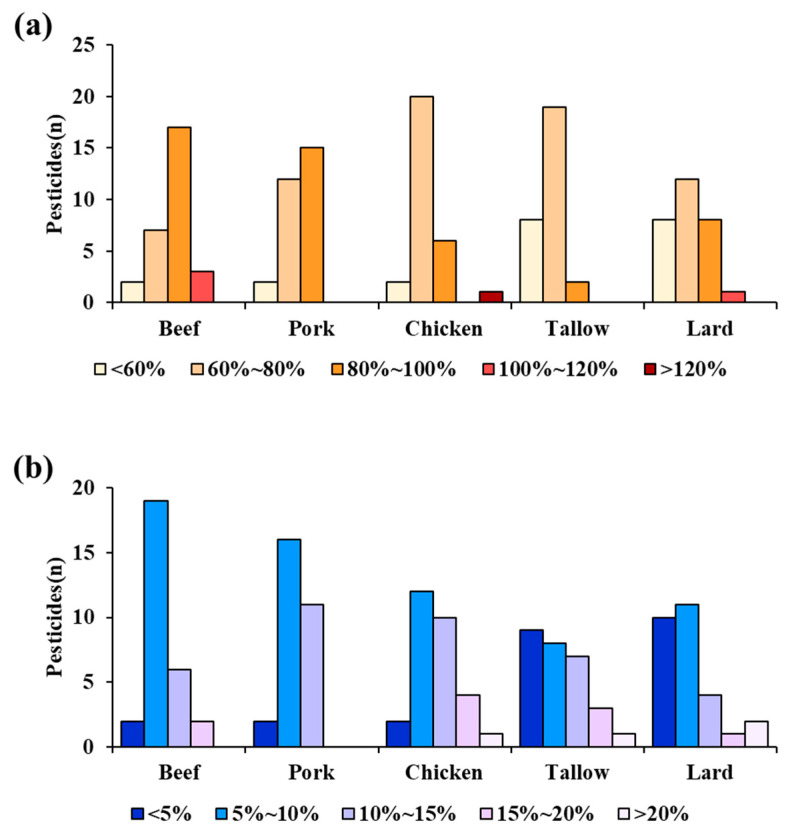
(**a**) Distribution of overall recoveries and (**b**) RSD (in percent, *n* = 5) of the 32 compounds in beef, pork, chicken, tallow, and lard samples, which were obtained using the developed method.

The distributions of recovery and RSD for all samples are shown in Figure 3a. For the beef, pork, and chicken samples, the recovery of the target compounds was 75–81% to satisfy the 70–120% criteria. In contrast, for the tallow and lard samples, the mean recovery was 63% and 66%, respectively. For the tallow and lard samples, the number of pesticides satisfying the mean recovery of <60% increased, showing a fall in recovery with increasing fat content. This result coincided with the findings of Hwang et al. [29] who reported that when the samples were divided according to fat content, those with a higher fat content showed a lower recovery, even with the use of an identical analytical method for residual pesticides. In particular, the mean recovery of chinomethionat, cyprodinil, pendimethalin, pentachloroaniline, and quintozene showed a sudden decrease from 67.6–96.8% in the beef, pork, and chicken samples to 11.3–45.0% in the tallow and lard samples. In addition, the log Pow of the five compounds was in the range of 3.8–5.4, indicating their nonpolarity. For tallow and lard with 100% fat content, the fat forms a layer instead of being completely dissolved in acetonitrile during extraction. The fat layer is highly nonpolar, and the five aforementioned compounds are highly likely to disperse in the fat layer. The five compounds in the fat layer decreased the extraction efficiency, which ultimately reduced the recovery of the compounds from the tallow and lard samples. Lehotay et al. [24] reported that lipids do not easily dissolve in acetonitrile, but the extracted fat forms a layer on the extraction solvent and emulsion surface. Nonpolar pesticides are dispersed in the undissolved fat layer to lower the recovery during extraction with acetonitrile. For food with a low fat content, the fat is dissolved in acetonitrile to achieve a high recovery.

The recovery of each pesticide sample in relation to the recovery criteria is shown in Table A3. At all spiking levels, the compounds with consistently low recovery rates were 3,5-dichloroaniline and dichlorobenil, with 3,5-dichloroaniline as a metabolite of vinclozoline displaying the lowest recovery at 11.5–20.2%. Vinclozoline belongs to the dicarboximide class of compounds, and the QuEChERS extraction could be applied to these compounds due to their molecular mass of 162.3 and log P_ow_ of 2.9. However, owing to the C_18_ SPE cartridge used in the cleanup step, the elution was shown to be poor due to the solvent extracting power and adsorbent properties. According to a study by Tsochatzis et al. [30], the recovery of 3,5-dichloroaniline through QuEChERS extraction was approximately 70%, which does not agree with the results reported in this study. In addition, dichlorobenil with log P_ow_ of 2.7 has shown to exhibit poor elution similar to that of 3,5-dichloroaniline, due to solvent properties.

#### 3.4.2. Precision

As shown in Figure 3b, the RSD is presented to evaluate the precision of the analytical method. Results showed that the RSD was only >20% for pendimethalin and quintozene in the tallow and lard samples. This is presumed to be due to the difference in the distribution efficiency based on log P_ow_ in the extraction process and recovery. Thus, only the sensitivity of the analytical method to 27 compounds in the beef, pork, and chicken samples, and 21 compounds in the tallow and lard samples out of the 32 compounds, has satisfied the Codex Alimentarius criteria. This suggests that the optimized analytical method developed in this study can be employed to determine such compounds with high accuracy, reproducibility, and efficiency. For most livestock products, the multi-residue analytical method for various pesticides is based on the QuEChERS method. Most studies on the multi-residue analytical method for various pesticides in livestock products applying GC-MS/MS, validated the analytical method using only one or two samples of livestock products such as chicken and milk [31,32,33]. In this study, five typical livestock commercial meat products, namely, beef, pork, chicken, tallow and lard, were selected to validate the analytical method and to investigate the potential accumulation of oil-soluble pesticides in the meat samples. Therefore, the results in this study serve as a significant reference for the potential application of the developed optimized multi-residue analytical method for the determination of the 32 compounds in livestock products.

### 3.5. Matrix Effect

**Figure 4:**
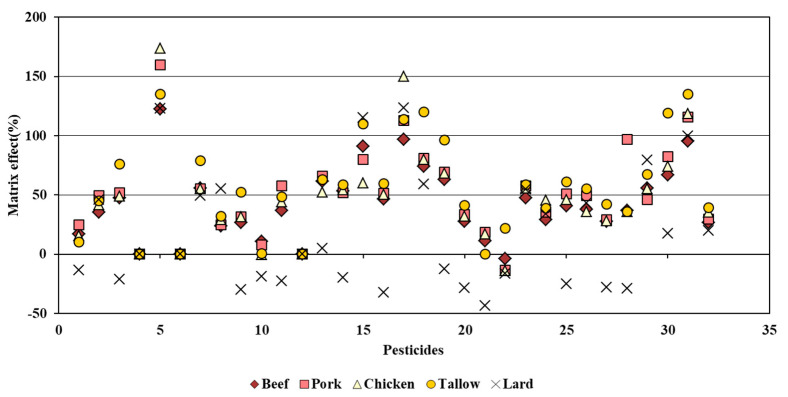
Matrix effects on the 32 compounds from beef, pork, chicken, tallow, and lard using the developed method.

**Figure A1:**
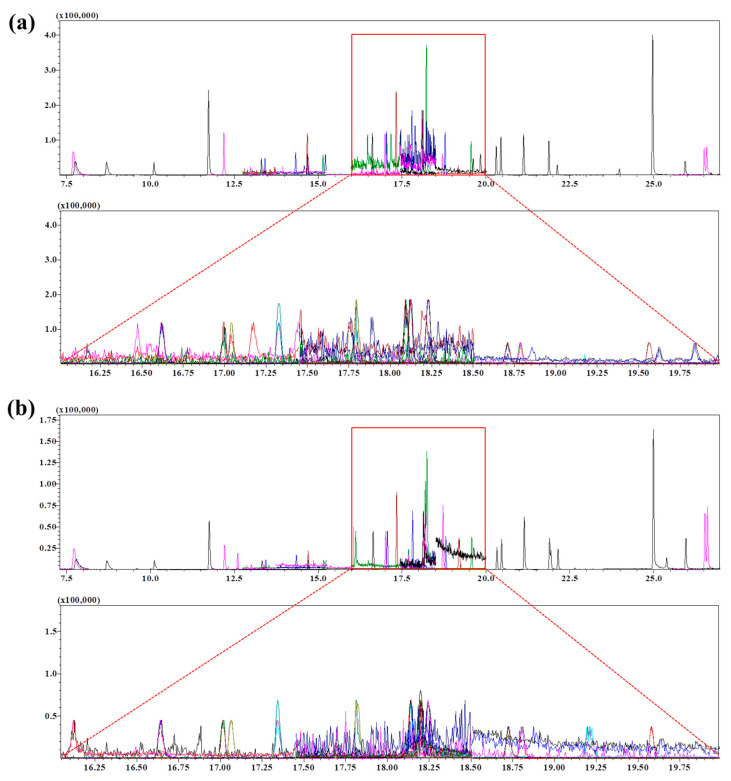
Comparison of the TICs for the 32 compounds in beef, which were obtained using the (**a**) dSPE, and (**b**) C_18_ SPE for the cleanup step.

**Figure A2:**
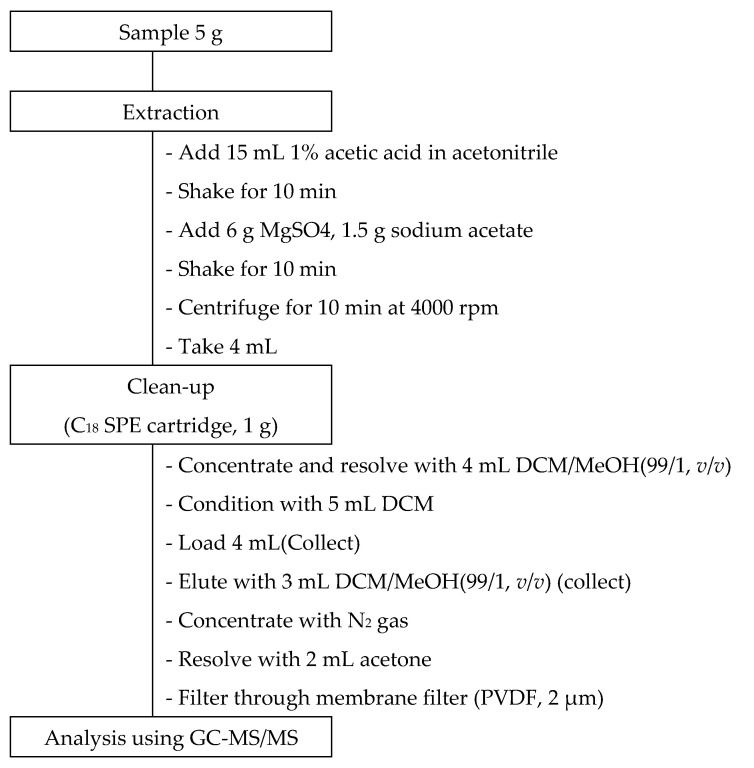
Flow chart of sample preparation for the multi-residue analysis method.

The ME on each compound using the analytical method developed in this study is shown in Figure 4. The estimated MEs were as follows: −3.9–122.5% for beef, −13.6–159.8% for pork, −14.1–173.9% for chicken, 0.4–135.2% for tallow, and −43.5–123.4% for lard. The number of pesticides satisfying the −50–50% criteria indicating a low ME [34] was *n* = 20 for beef, *n* = 15 for pork, *n* = 18 for chicken, *n* = 13 for tallow, and *n* = 23 for lard. All but one or two compounds in the four sample types excluding lard showed a phenomenon of ion enhancement, where the reactivity of the signal increased. This is a known characteristic of GC-MS/MS. The matrix binds to the active site of the GC column, and the consequently unbound target compound is eluted to increase the signal [35]. Among the target compounds, chinomethionat showed the highest ME for the five sample types, which was at 122.5–173.9%. Nine out of the 32 compounds including chinomethionat with ME > 50% were shown to be relatively nonpolar with log P_ow_ of 3.3–6.24. These compounds also included those based on carbamate, amine, triazole, pyrethroid, amide, and carboxamide, which are reported to show the enhancement phenomenon due to the ME in GC-MS/MS [36]. Meanwhile, for the lard sample, 50% of the target compounds showed ion suppression with a negative value of ME. This is considered to be due to the extraction of certain compounds, including nonvolatile ones in the lard, which accumulated inside the GC column to create new active sites for binding of the target compounds and thus decreasing the signal [37].

### 3.6. Application and Monitoring of Meat Products

In order to evaluate whether the developed analytical method is applicable in the actual field, monitoring was performed on 269 meat products (64 beef, 71 pork, 70 chicken, 32 mammalian offal, and 32 poultry offal) collected from the domestic markets in Korea with the developed analytical method. As shown in Table 2, a total of four pesticides were detected: atrazine, difenoconazole, pendimethalin, and propiconazole. In beef and pork samples, difenoconazole and propiconazole were detected in one sample, respectively, and their concentrations were below the MLOQ. In chicken samples, difenoconazole and propiconazole were detected in two samples, and the concentrations were 11 and 5 µg/kg. Propiconazole detected in chicken samples was lower than the Codex MRLs (Propiconazole: 0.01 mg/kg in chicken). On the other hand, difenoconazole exceeded the Codex MRLs (0.01 mg/kg). Offal with a relatively higher fat content than muscle was additionally purchased and monitored. No pesticides were detected in mammalian offal. However, it was confirmed that atrazine and pendimethalin were detected only in the gizzard of the poultry offal.

## 4. Conclusions

In summary, an analytical method for the determination of pesticides was proposed for the rapid monitoring of the 29 pesticides and related metabolites in livestock products. In addition, the proposed analytical method was validated and optimized in accordance with Codex guidelines. The optimized method will be a novel method due to its robustness, simplicity and short operation time. Furthermore, a C_18_ SPE cartridge was considered as the effective adsorbent to remove the coextracts, and thus reflect only the characteristics of the samples from livestock products. The recovery test also confirmed its high accuracy, precision, and reproducibility during analysis of the 32 compounds, including pesticides and metabolites in the different meat products. Therefore, this analytical method is predicted to contribute to the development of a consistent, accurate, and reliable monitoring system with a high rate and efficiency for the determination of target pesticide in livestock products. However, the ME is a critical concern during development of a simultaneous analytical method for pesticides in meat products. Furthermore, future studies should focus on the reduction of ME for a more extensive application of the developed analytical method for the simultaneous analysis of residual pesticides in livestock products, in light of the findings of this investigation.

## Figures and Tables

**Table 1:** Method limit of quantification and linearity for the 32 compounds in the samples.

No.	Compound	MLOQ (mg/kg)	Linearity (*R*^2^)				
			Beef	Pork	Chicken	Tallow	Lard
1	3,5-Dichloroaniline	0.005	0.9997	0.9995	0.9995	0.9999	0.9992
2	Alachlor	0.005	0.9997	0.9995	0.9995	0.9990	0.9954
3	Atrazine	0.005	0.9993	0.9992	0.9992	0.9996	0.9992
4	Captan	N.C. ^1^	N.C. ^1^	N.C. ^1^	N.C. ^1^	N.C. ^1^	N.C. ^1^
5	Chinomethionat	0.005	0.9992	1.0000	1.0000	0.9999	0.9996
6	Chlorothalonil	N.C.	N.C.	N.C.	N.C.	N.C.	N.C.
7	Chlorpropham	0.005	0.9984	0.9983	0.9983	1.0000	0.9999
8	Cyanazine	0.005	0.9996	0.9996	0.9996	0.9999	0.9977
9	Cyprodinil	0.005	0.9997	0.9998	0.9998	1.0000	0.9994
10	Dichlorobenil	0.005	0.9999	0.9991	0.9991	0.9999	0.9986
11	Difenoconazole	0.005	0.9994	0.9977	0.9977	1.0000	0.9998
12	Dimethipin	N.C.	N.C.	N.C.	N.C.	N.C.	N.C.
13	Diphenylamine	0.005	0.9997	0.9993	0.9993	1.0000	0.9997
14	Epoxiconazole	0.005	0.9994	0.9997	0.9997	1.0000	0.9997
15	Esfenvalerate	0.005	0.9995	0.9999	0.9999	0.9990	0.9998
16	Fenamidone	0.005	0.9993	0.9997	0.9997	1.0000	0.9997
17	Fenoxanil	0.02	0.9967	0.9967	0.9967	0.9971	0.9945
18	Flumioxazin	0.01	0.9989	0.9994	0.9994	0.9999	0.9999
19	Fluopyram	0.005	0.9997	0.9996	0.9996	0.9984	0.9996
20	Flusilazole	0.005	0.9992	0.9999	0.9999	0.9962	0.9989
21	Flutriafol	0.005	0.9991	0.9996	0.9996	N.C.	0.9987
22	PAM	0.005	0.9990	0.9992	0.9992	1.0000	0.9992
23	Pendimethalin	0.005	0.9996	0.9996	0.9996	0.9998	0.9998
24	Pentachloroaniline	0.005	0.9990	0.9996	0.9996	1.0000	0.9994
25	Penthiopyrad	0.005	0.9995	0.9998	0.9998	0.9999	0.9992
26	Phthalide	0.005	0.9989	0.9992	0.9992	0.9999	0.9997
27	Picoxystrobin	0.005	0.9996	0.9990	0.9990	0.9998	0.9983
28	Propiconazole	0.005	0.9994	0.9995	0.9995	1.0000	0.9998
29	Quintozene	0.01	0.9980	0.9988	0.9988	0.9992	0.9989
30	Simazine	0.005	0.9995	0.9994	0.9994	0.9993	0.9995
31	Thifluzamide	0.005	0.9996	0.9999	0.9999	0.9984	0.9997
32	Vinclozoline	0.005	0.9996	0.9978	0.9978	0.9999	0.9999

^1.^ N.C.: not calculated.

**Table 2:** A summary of monitoring of 32 analyzed compounds for 269 commercial meat products in Korea, by the developed method for the detected number and related pesticides and their respective concentrations.

Sample Origin	No. of Samples Analyzed	No. of Detected Samples	Part of the Detected Sample	Detected Compounds	Concentrations (µg/kg)
Beef	64	1	Ground beef	Difenoconazole	<MLOQ
Pork	71	1	Pork belly	Propiconazole	<MLOQ
Chicken	70	2	Chicken chest	Difenoconazole	11
			Drumstick	Propiconazole	5
Mammalian offal	32	-	-	-	-
Poultry offal	32	2	Gizzard	Atrazine	14
			Gizzard	Pendimethalin	5

## Data Availability

The data presented in this study are available on request from the corresponding author.

## Supplementary Materials

The following supporting information can be downloaded at: https://www.mdpi.com/article/10.3390/foods11192930/s1, Table S1: Physico-chemical properties and MRL of analytical pesticides in beef, pork, and chicken; Table S2: Commodity groups and representative commodities.

## Appendix A

**Table A1:** GC-MS/MS parameters and retention time optimized for the determination of 32 compounds.

No.	Pesticide	*t*_R_	Precursor Ion > Product Ion (CE, eV)	
			Quantifier Ion	Qualifier Ion
1	3,5-dichloroaniline	8.63	146.0 > 118.1 (9)	146.0 > 131.1 (9)
2	Alachlor	15.3	149.0 > 105.0 (5)	114.0 > 79.0 (15)
3	Atrazine	13.43	206.0 > 148.1 (15)	206.0 > 121.1 (24)
4	Captan	17.5	264.0 > 167.8 (25)	266.0 > 132.8 (35)
5	Chinomethionat	17.86	127.0 > 65.0 (21)	127.0 > 92.1 (15)
6	Chlorothalonil	14.01	224.0 > 208.0 (25)	224.0 > 118.0 (40)
7	Chlorpropham	12.19	171.0 > 100.1 (24)	171.0 > 136.0 (12)
8	Cyanazine	14.32	323.0 > 265.0 (15)	325.0 > 267.0 (18)
9	Cyprodinil	17.11	323.0 > 265.0 (15)	325.0 > 267.0 (18)
10	Dichlorobenil	7.69	118.0 > 58.1 (5)	118.0 > 90.1 (5)
11	Difenoconazole 1	26.62	169.0 > 167.1 (27)	169.0 > 66.1 (24)
12	Difenoconazole 2	26.69	192.0 > 138.0 (15)	192.0 > 111.0 (25)
13	Dimethipin	13.4	197.0 > 141.1 (15)	197.0 > 115.1 (25)
14	Diphenylamine	11.75	189.0 > 125.0 (15)	293.0 > 198.0 (15)
15	Epoxiconazole	21.21	354.0 > 326.0 (10)	354.0 > 176.0 (15)
16	Esfenvalerate	25.1	173.0 > 145.0 (15)	223.0 > 196.0 (15)
17	Fenamidone	21.93	233.0 > 165.2 (20)	233.0 > 152.2 (15)
18	Fenoxanil	19.27	193.0 > 177.1 (10)	177.0 > 101.1 (15)
19	Flumioxazin	26.02	252.1 > 162.1 (10)	252.0 > 208.0 (5)
20	Fluopyram	17.39	177.0 > 101.0 (20)	302.0 > 152.0 (5)
21	Flusilazole	18.85	335.0 > 173.0 (10)	335.0 > 303.0 (10)
22	Flutriafol	18.27	173.0 > 145.0 (20)	259.0 > 69.0 (10)
23	PAM	7.6	173.0 > 145.0 (20)	259.0 > 69.0 (10)
24	Pendimethalin	17.09	265.0 > 236.8 (12)	265.0 > 194.0 (15)
25	Pentachloroaniline	14.72	201.0 > 173.1 (6)	201.0 > 186.1 (6)
26	Penthiopyrad	19.63	194.0 > 125.0 (20)	166.0 > 125.0 (15)
27	Phthalide	16.66	212.0 > 172.0 (15)	212.0 > 145.0 (24)
28	Picoxystrobin	18.21	161.0 > 99.0 (25)	161.0 > 90.1 (20)
29	Propiconazole 1	20.4	215.0 > 58.0 (20)	200.0 > 104.0 (20)
30	Propiconazole 2	20.54	212.0 > 123.1 (20)	212.0 > 151.1 (15)
31	Quintozene	13.47	265.0 > 194.1 (20)	265.0 > 192.1 (20)
32	Simazine	13.3	123.0 > 95.1 (15)	219.0 > 123.1 (15)
33	Thifluzamide	18.75	238.0 > 103.1 (25)	268.0 > 180.1 (25)
34	Vinclozoline	15.21	243.0 > 214.9 (15)	243.0 > 179.0 (30)

**Table A2:** Analytical conditions for residue analysis using GC-MS/MS.

**GC Conditions**			
Model	GC 2010 Plus (Shimadzu, Japan)		
Column	ZB-5MS Plus [30 m(L) × 0.25 mm(i.d.), 0.25 μm]		
Injection mode	Splitless		
Carrier gas	He, 1.5 mL/min		
Oven temp.	Rate (°C/min)	Final Temp (°C)	Hold Time (min)
	0	90	3.0
	20.0	120	0
	8.0	300	3.0
Injection volume	2 μL		
**Tandem MS conditions**			
Model	GC-TQ8050 (Shimadzu, Japan)		
Ionization mode	Electron ionization (EI), −70 eV		
Source temp.	250 °C		
Interface temp.	300 °C		
Q2 collision gas	Argon		
Collision pressure	1.50 mTorr		

**Table A3:** Average recovery, inter-day precision, and intra-day precision of the 32 compounds in the beef, pork, chicken, tallow and lard samples.

Compounds	Spiked Level (µg/kg)	Beef (*n* = 5)			Pork (*n* = 5)			Chicken (*n* = 5)			Tallow (*n* = 5)			Lard (*n* = 5)		
		Average Recovery (%)	Intra-day RSD (%)	Inter-Day RSD (%)	Average Recovery (%)	Intra-Day RSD (%)	Inter-Day RSD (%)	Average Recovery (%)	Intra-Day RSD (%)	Inter-Day RSD (%)	Average Recovery (%)	Intra-Day RSD (%)	Inter-Day RSD (%)	Average Recovery (%)	Intra-Day RSD (%)	Inter-Day RSD (%)
3,5-dichloroaniline	5	11.5	25.7	9.9	45.4	2.6	5.7	15.7	21.0	0.3	19.9	15.2	1.8	15.9	18.9	3.0
	10	14.6	19.3	5.6	34.5	13.1	10.1	12.9	23.4	8.5	15.3	12.9	5.1	25.8	12.8	6.9
	100	20.1	10.2	6.9	27.2	3.9	9.3	28.6	6.3	0.3	27.0	14.6	3.8	30.9	7.0	3.5
	500	20.2	4.7	3.1	25.4	14.9	3.3	24.3	5.9	9.4	28.2	5.1	7.1	31.3	5.5	9.6
Alachlor	5	70.3	25.0	3.0	62.7	4.5	6.2	76.7	15.3	1.8	69.2	9.7	2.3	69.9	21.7	0.8
	10	70.9	19.3	1.5	65.6	12.3	1.5	83.9	4.4	6.5	60.5	15.4	8.2	70.5	13.9	6.1
	100	73.6	3.2	2.1	79.5	1.8	6.1	90.1	4.9	8.5	73.4	9.3	3.3	72.6	5.1	2.9
	500	84.4	12.8	5.7	78.0	4.6	5.2	98.4	3.9	5.7	74.3	10.7	7.3	75.0	2.4	9.1
Atrazine	5	85.1	13.7	7.6	104.6	6.0	0.8	68.4	26.4	9.2	65.6	15.6	8.1	62.4	17.2	1.2
	10	69.4	18.2	1.1	74.4	23.4	6.4	68.8	15.4	6.7	68.3	8.7	9.9	61.2	8.6	0.3
	100	80.9	3.4	5.3	82.0	2.3	5.0	88.4	2.3	3.4	82.5	5.3	7.8	73.4	1.5	3.4
	500	85.5	9.6	5.2	83.0	8.0	9.5	79.8	1.0	9.7	89.3	3.4	7.3	77.1	6.2	2.4
Chinomethionat	5	113.1	20.5	0.6	71.1	1.3	3.8	66.1	21.2	7.3	27.6	21.6	7.9	17.6	18.6	5.6
	10	55.4	16.2	8.4	50.8	19.7	0.1	61.8	21.6	8.7	25.2	14.4	10.2	33.9	18.7	1.8
	100	67.1	11.9	1.5	79.9	4.4	2.6	71.6	9.2	5.1	29.1	7.1	2.2	33.1	14.4	3.3
	500	69.8	22.5	7.2	77.5	11.5	3.4	70.8	17.6	6.4	29.4	4.3	3.5	33.0	6.5	8.1
Chlorpropham	5	111.1	4.5	9.2	88.4	20.7	1.2	62.5	15.0	2.9	61.2	8.6	4.6	87.6	3.2	4.8
	10	89.5	8.8	3.4	68.8	14.1	0.8	73.7	17.9	2.3	67.3	3.8	3.1	86.4	6.6	2.5
	100	75.1	7.4	3.6	73.4	7.3	7.8	78.3	5.8	9.9	77.3	6.1	6.7	78.3	4.3	10.2
	500	77.2	13.5	5.5	77.5	5.1	2.6	78.4	19.2	7.0	73.1	1.4	8.5	77.7	1.7	3.2
Cyanazine	5	105.9	9.4	8.5	87.8	11.7	1.0	64.0	22.0	1.4	64.5	2.9	3.6	65.5	8.3	6.7
	10	87.5	9.4	2.3	75.8	25.7	3.7	75.0	19.7	4.3	68.6	1.5	4.7	74.5	16.4	7.3
	100	90.4	0.8	2.2	88.7	3.7	3.5	91.8	3.0	9.4	80.9	8.6	2.4	89.8	4.7	1.7
	500	90.4	11.1	8.5	92.8	5.5	9.8	89.3	10.0	7.9	84.5	5.6	5.3	91.7	1.5	9.4
Cyprodinil	5	63.1	19.5	2.7	71.2	1.4	1.7	66.7	3.7	6.2	31.1	17.1	3.0	27.2	25.6	1.3
	10	62.5	7.7	8.6	62.5	14.3	4.5	73.0	12.1	9.9	12.3	16.7	2.0	43.7	4.3	2.6
	100	70.8	3.6	4.7	70.0	2.3	3.0	77.7	1.2	2.8	33.7	15.4	7.2	53.4	3.6	2.0
	500	75.7	4.6	10.0	73.0	4.4	7.7	73.8	5.0	3.4	29.8	5.2	1.3	55.7	3.3	4.4
Dichlorobenil	5	40.4	9.1	0.2	44.8	7.3	7.9	45.8	9.6	8.2	33.6	11.5	9.6	27.7	20.0	6.0
	10	38.0	14.7	0.9	28.9	17.4	9.7	24.8	28.8	6.7	29.6	10.6	2.3	43.7	7.3	9.0
	100	50.6	9.8	5.4	27.9	16.3	7.9	37.5	9.2	4.3	40.2	12.3	4.3	51.1	8.2	3.1
	500	52.7	19.5	7.4	52.3	19.8	1.3	51.4	16.4	4.4	37.0	6.9	4.1	60.5	12.0	6.6
Difenoconazole	5	95.5	5.7	9.3	113.0	1.1	0.0	65.1	7.3	3.7	66.0	3.2	2.8	60.5	3.4	8.2
	10	84.1	7.5	5.2	101.1	14.0	3.6	70.8	6.2	1.2	61.6	1.0	3.2	65.1	4.6	4.9
	100	85.2	2.7	3.6	76.9	1.7	6.7	85.5	3.1	7.2	85.6	8.4	3.6	76.7	2.7	6.3
	500	86.0	6.0	0.5	82.4	2.0	5.4	83.9	6.5	1.6	88.9	1.3	8.7	80.6	1.7	0.4
Diphenylamine	5	71.6	3.4	10.0	64.2	3.0	3.0	76.9	7.1	9.2	60.3	3.7	7.3	67.7	4.8	7.9
	10	78.3	7.8	8.1	76.9	12.5	9.1	71.4	7.7	0.0	67.4	5.3	1.8	68.3	2.4	0.7
	100	77.5	3.6	0.7	71.1	2.1	1.1	72.5	0.5	1.6	71.5	5.7	6.1	80.9	2.7	3.2
	500	76.2	5.1	1.2	76.1	3.8	8.8	79.3	8.0	9.2	74.4	4.4	0.2	86.9	3.0	5.6
Epoxiconazole	5	94.5	10.4	0.5	94.2	3.4	8.0	60.4	7.4	4.8	68.4	6.5	2.2	71.6	3.4	6.6
	10	83.1	7.6	0.0	78.9	21.1	2.5	68.9	8.6	8.4	62.7	1.9	9.0	80.3	2.8	1.1
	100	82.7	3.0	7.1	83.4	2.4	4.3	85.6	3.0	3.2	80.9	9.7	3.7	82.3	2.5	6.7
	500	86.3	8.3	6.1	86.0	4.5	4.3	83.6	6.5	8.0	88.0	0.3	1.0	85.2	3.8	4.5
Esfenvalerate	5	117.5	5.8	8.0	83.3	11.9	8.0	65.2	7.0	6.7	73.5	5.1	7.9	63.6	3.3	1.4
	10	88.5	9.7	5.2	66.3	18.4	0.9	70.8	9.1	4.4	67.4	3.1	4.7	67.5	3.5	1.4
	100	83.0	5.6	6.3	74.0	4.5	3.1	81.4	8.0	2.6	77.7	7.1	4.0	71.2	1.5	6.1
	500	84.4	12.5	0.1	78.7	10.5	3.6	79.8	7.5	10.0	74.7	2.8	10.1	73.3	3.6	3.7
Fenamidone	5	88.9	11.9	4.0	82.9	7.8	5.2	63.6	3.3	2.3	79.3	5.6	4.0	86.0	5.6	6.1
	10	78.9	9.7	0.5	73.6	21.8	9.7	69.1	10.7	1.9	74.7	1.5	0.2	90.3	2.0	6.0
	100	84.3	3.1	4.6	83.3	1.9	8.8	88.5	1.9	3.0	87.3	9.9	7.7	96.0	2.1	1.9
	500	88.6	6.5	9.0	86.0	3.4	9.2	84.1	4.0	7.8	84.9	1.3	0.1	99.9	2.9	1.4
Fenoxanil	5	120.8	12.4	10.0	115.7	9.0	5.7	135.8	12.4	8.1	60.1	16.9	10.0	132.3	19.6	9.5
	10	135.4	11.9	1.9	49.5	3.3	0.5	132.3	11.5	0.6	64.6	11.8	7.6	121.5	17.0	6.6
	100	91.3	9.2	9.3	71.8	21.4	6.4	106.0	10.7	3.9	60.2	1.9	7.4	71.9	15.9	0.3
	500	81.9	26.7	0.6	87.5	26.2	8.7	122.6	13.2	4.0	69.0	11.8	5.5	78.6	2.9	9.4
Flumioxazin	5	117.6	5.6	9.5	115.7	13.3	8.3	69.6	13.0	3.4	84.9	16.5	7.7	86.7	7.1	7.9
	10	119.1	10.3	2.1	91.0	21.8	7.3	77.1	19.3	7.9	76.9	11.5	1.2	82.2	10.7	7.3
	100	100.8	5.4	3.0	86.6	2.3	1.5	90.9	5.0	8.8	85.5	8.5	1.8	83.6	0.6	0.4
	500	96.1	15.7	6.6	91.6	10.6	3.1	91.6	12.2	9.3	82.5	3.3	8.8	86.9	3.7	4.5
Fluopyram	5	114.4	9.1	10.1	97.2	4.2	8.2	63.7	9.2	5.4	69.5	12.1	10.1	70.4	3.5	1.0
	10	93.3	7.8	5.5	84.5	18.1	8.5	67.9	11.6	8.1	73.5	0.7	6.9	81.1	2.1	1.4
	100	86.4	3.2	7.8	82.9	1.5	3.0	89.6	2.1	6.1	83.8	10.7	4.7	87.7	3.3	5.9
	500	91.1	6.8	9.3	86.3	3.7	7.5	84.3	5.6	4.6	80.7	1.2	4.4	90.0	3.6	5.4
Flusilazole	5	90.3	9.8	2.6	93.7	3.5	8.1	62.0	16.1	0.5	61.1	8.8	7.3	67.5	9.2	3.1
	10	85.5	18.1	2.5	81.4	19.5	4.7	66.6	12.5	2.7	60.7	3.1	8.6	74.9	5.9	4.7
	100	85.9	3.1	7.0	86.2	0.4	6.9	88.7	2.8	5.4	74.1	12.6	5.5	86.3	3.0	7.7
	500	90.3	7.7	8.4	89.0	3.6	6.7	84.1	5.4	4.9	72.5	0.7	1.8	89.5	3.2	8.5
Flutriafol	5	116.0	6.4	9.5	110.6	2.3	0.7	64.8	18.7	9.8	N.D.		8.0	N.D.		
	10	87.7	5.6	9.9	84.3	11.3	8.7	137.5	76.2	6.7	N.D.		1.1	N.D.		
	100	83.0	5.0	6.3	84.0	2.7	1.8	88.0	3.8	2.8	N.D.		0.3	N.D.		
	500	86.6	5.9	4.6	86.2	6.3	8.8	87.3	10.8	9.1	N.D.		8.6	N.D.		
PAM	5	102.3	4.0	3.7	106.3	4.9	5.4	100.3	9.8	7.9	62.7	6.5	9.8	76.3	2.9	3.1
	10	74.1	19.3	3.4	85.4	18.5	8.5	78.9	4.7	5.6	77.3	1.9	9.2	74.1	7.0	3.3
	100	70.9	14.1	0.3	86.8	5.8	4.8	71.6	9.7	3.4	70.6	8.0	1.8	73.3	8.7	9.5
	500	72.6	6.0	0.7	71.5	17.2	7.3	73.0	11.8	3.2	79.2	1.5	4.4	78.3	6.1	4.8
Pendimethalin	5	119.6	6.7	4.2	95.7	14.1	0.8	61.7	18.4	9.8	22.5	16.4	7.5	37.5	22.1	7.7
	10	87.7	6.8	3.8	62.0	18.3	7.6	63.5	16.9	8.2	21.7	11.1	8.7	48.7	19.0	3.4
	100	78.2	4.2	3.6	79.0	3.0	2.6	86.5	6.6	5.5	30.9	14.6	0.9	30.5	12.6	1.3
	500	72.6	20.6	4.5	79.1	15.2	1.5	76.5	12.6	6.0	35.4	10.9	4.1	31.2	5.9	4.9
Pentachloroaniline	5	70.7	3.1	0.7	68.8	4.5	2.4	68.5	5.1	2.8	26.0	21.5	0.8	37.1	13.9	3.3
	10	60.8	11.4	7.5	65.4	15.9	6.2	73.1	3.4	3.3	34.3	6.8	8.3	42.5	9.9	5.7
	100	81.2	2.0	3.9	75.8	0.4	7.9	77.2	1.5	1.3	39.7	9.8	3.5	42.7	3.0	3.2
	500	75.0	5.6	7.3	76.6	3.6	8.5	77.8	2.7	8.3	42.9	7.5	2.4	45.6	4.4	3.9
Penthiopyrad	5	104.4	3.7	9.0	93.1	1.3	1.2	69.5	18.7	9.9	68.8	12.8	2.2	65.5	3.1	1.9
	10	83.3	9.8	7.8	77.1	17.5	9.8	79.7	6.9	3.7	70.0	2.3	5.4	79.7	4.9	4.4
	100	89.2	2.8	3.9	83.1	1.6	3.5	89.5	1.8	1.9	88.9	10.4	3.5	88.1	3.1	3.9
	500	92.2	7.6	2.0	84.8	3.5	3.4	84.9	5.4	7.9	83.5	3.8	2.1	91.1	0.6	8.3
Phthalide	5	103.4	14.0	3.9	109.8	16.6	4.3	65.2	13.9	0.4	70.0	10.4	0.3	62.1	9.9	3.6
	10	104.3	8.2	6.9	72.5	16.0	6.2	74.5	21.6	3.6	73.6	4.9	5.0	70.3	9.1	4.9
	100	88.2	5.3	6.6	71.8	5.8	8.5	81.4	7.4	3.6	88.9	7.0	4.2	83.1	5.6	7.9
	500	95.3	15.9	2.1	80.8	14.6	2.7	84.5	13.1	4.8	81.4	1.4	5.0	86.6	5.0	8.1
Picoxystrobin	5	80.1	5.7	4.7	83.4	2.4	6.6	68.5	17.5	9.2	73.6	1.6	6.8	64.1	13.3	5.0
	10	73.8	5.7	9.9	70.5	13.5	0.8	65.1	17.0	9.7	60.5	3.9	5.0	83.4	6.7	9.4
	100	85.3	4.6	9.9	84.8	4.6	4.6	90.4	2.2	7.3	74.8	9.9	4.1	88.0	2.5	4.7
	500	89.1	6.1	0.2	86.7	2.1	5.0	86.6	8.0	3.2	73.1	2.0	1.5	93.3	2.1	9.2
Propiconazole	5	102.1	7.5	10.2	68.8	2.7	10.0	63.1	6.3	4.5	60.9	12.8	8.0	66.4	6.6	2.7
	10	85.7	4.3	0.2	70.3	14.9	9.8	63.1	13.1	7.7	60.6	3.1	9.1	67.8	2.6	9.4
	100	80.6	4.2	7.7	77.9	1.6	3.2	87.8	2.0	5.8	86.0	8.3	6.4	80.6	3.6	3.8
	500	86.2	5.3	7.2	76.8	2.6	8.5	82.8	6.5	1.7	88.7	1.7	6.6	84.0	3.5	7.3
Quintozene	5	111.3	10.1	4.1	63.8	2.0	0.9	64.4	11.9	4.8	N.D.		3.5	N.D.		
	10	89.5	4.2	0.8	63.1	27.8	0.6	68.3	20.6	9.4	N.D.		2.0	35.1	36.6	9.3
	100	91.2	6.0	8.2	79.2	4.3	4.8	72.0	3.9	0.8	15.4	30.7	7.7	26.8	9.8	0.8
	500	95.3	11.5	4.3	74.7	10.3	3.9	75.6	16.9	8.9	7.2	57.4	0.7	32.4	39.0	2.9
Simazine	5	82.1	14.0	4.2	64.9	0.9	9.4	69.3	2.0	6.8	71.9	24.3	2.6	70.4	9.9	4.9
	10	78.8	13.5	4.6	60.1	12.3	5.0	73.9	15.9	0.0	75.9	5.8	6.9	79.5	4.2	0.1
	100	81.7	3.2	10.1	80.9	5.7	8.2	86.9	4.7	9.4	88.2	8.0	9.7	74.8	3.2	0.7
	500	84.1	7.9	4.7	81.9	1.0	9.9	83.7	5.0	6.7	82.2	6.9	3.9	80.9	7.0	5.5
Thifluzamide	5	115.3	6.1	8.2	98.2	7.6	7.1	65.2	7.4	8.5	60.5	9.0	9.2	66.8	10.5	3.6
	10	111.7	8.1	3.8	81.8	27.1	0.1	71.1	9.1	9.8	70.1	4.2	1.4	80.7	1.7	1.5
	100	96.0	6.1	6.8	84.4	2.1	0.4	88.9	3.7	0.8	80.7	10.7	8.1	88.3	4.5	1.0
	500	100.6	9.2	4.0	87.8	5.4	1.9	83.8	3.8	0.5	85.3	1.6	9.3	90.4	2.2	1.8
Vinclozoline	5	69.3	9.4	6.0	69.7	2.2	7.8	69.3	11.8	1.4	80.0	9.1	1.2	64.4	9.8	3.4
	10	69.7	2.4	6.4	77.3	17.9	4.7	65.2	19.0	3.0	65.1	14.4	7.9	63.9	4.2	2.2
	100	76.3	2.3	2.6	76.9	1.5	0.5	85.6	1.7	5.3	71.2	9.6	1.7	76.7	3.0	0.1
	500	85.6	5.2	5.3	78.0	1.9	6.8	80.1	8.5	0.4	72.3	9.4	5.9	82.0	5.0	6.0
