# Supplementary material for: Optimization of a Multi-Residue Analytical Method during Determination of Pesticides in Meat Products by GC-MS/MS
Source: Foods. 2022 Sep 20;11(19):2930. doi: 10.3390/foods11192930 (PMC9563028; doi:10.3390/foods11192930)
Supplement: Supplementary file 1 [file foods-11-02930-s001.zip › foods-1875979-supplementary.pdf]

Table S1. Physico-chemical properties and MRL of analytical pesticides in beef, pork, and chicken

**No.**	Pesticide	**Substance group**	**Uses**	**log P_{ow}**	**Vapour pressure (mPa, 20°C)**	**Dissociation constant (pKa, 25°C)**	**Water solubility (mg/L, 20°C)**	**Molecular mass**	**MRL(mg/kg)**					
									**Codex**			**MFDS**		
									**Beef**	**Pork**	**Chicken**	**Beef**	**Pork**	**Chicken**
1	Alachlor	Chloroacetamide	Herbicide	3.09	2.9	0.62	240	269.77	-	-	-	-	-	-
2	Atrazine	Triazine	Herbicide	2.7	0.04	1.7	35	215.68	-	-	-	-	-	-
3	Captan[)]	Phthalimide	Fungicide	2.5	4.2×10^{-3}	No dissociation	5.2	300.61	-	-	-	-	-	-
4	Chinomethionate	Carbamate	Fungicide, Insecticide	3.78	0.03	-5.28	1	234.3	-	-	-	0.05	0.05	-
5	Chlorothalonil	Chloronitrile	Fungicide	2.94	0.08	No dissociation	0.81	265.91	0.02	0.02	0.01	-	-	-
6	Chlorpropham	Carbamate	PGR[a)], Herbicide	3.76	24	No dissociation	110	213.66	0.1	-	-	-	-	-
7	Cyanazine	Triazine	Herbicide	2.1	2.13×10^{-4}	12.9	171	240.69	-	-	-	-	-	-
8	Cyprodinil	Aminopyrimidine	Fungicide	4	0.51	4.44	13	225.29	0.01	0.01	0.01	-	-	-
9	Dichlobenil	Benzonitrile	Herbicide	2.7	2.9	0.62	240	269.77	0.01	0.01	0.03	-	-	-
10	Difenoconazole	Triazole	Fungicide	4.36	3.33×10^{-5}	1.07	15	406.26	0.2	0.2	0.01	-	-	-
11	Dimethipin	Sulfone	PGR	-0.17	0.05	10.9	1843	210.27	0.01	0.01	0.01	0.01	0.01	0.01
12	Diphenylamine	Amine	Fungicide	3.82	0.85	1.03	25.8	169.23	0.01	-	-	0.01(f)	-	-
13	Epoxiconazole	Triazole	Fungicide	3.3	0.01	No dissociation	7.1	329.76	-	-	-	-	-	-
14	Esfenvalerate	Pyrethroid	Insecticide	6.24	1.17×10^{-9}	No dissociation	0.001	419.91	-	-	0.01	-	-	-
15	Fenamidone	Imidazole	Fungicide	2.8	3.4×10^{-4}	No dissociation	7.8	311.4	0.01	0.01	0.01	-	-	-
16	Fenoxanil	Amide	Fungicide	3.53	2.1×10^{-4}	11.44	30700	329.22	-	-	-	-	-	-

17	Flumioxazin	N-phenylphtalamides	Herbicide	2.55	0.32	No dissociation	0.786	354.33	0.02	0.02	0.02	-	-	-
18	Fluopyram	Benzamide	Fungicide	3.3	1.2×10^{-3}	No dissociation	16	396.76	1.5	1.5	1.5	-	-	-
19	Flusilazole	Triazole	Fungicide	3.87	0.04	2.5	4.19	315.39	1	1	0.2	0.01	-	0.01
20	Flutriafol	Triazole	Fungicide	2.3	4.0×10^{-4}	2.3	95	301.29	0.02	0.02	0.01	-	-	-
21	Pendimethalin	Dinitroaniline	Herbicide	5.4	3.34	2.8	0.33	281.31	0.2	0.2	0.01	-	-	-
22	Penthiopyrad	Carboxamide	Fungicide	4.62	6.43×10^{-3}	10	1.375	359.4	0.04	0.04	0.03	-	-	-
23	Phthalide	Unclassified	Fungicide	3.01	3.0×10^{-3}	-	2.5	271.91	-	-	-	-	-	-
24	Picoxystrobin	Strobilurin	Fungicide	3.6	5.5×10^{-3}	No dissociation	3.1	367.32	0.02	0.02	0.01	-	-	-
25	Propiconazole	Triazole	Fungicide	3.72	0.06	1.09	150	342.22	0.01	0.01	0.01	0.05	0.05	0.05
26	Quintozene	Chlorophenyl	Fungicide	4.46	12.7	-	0.44	295.3	-	-	0.1	-	-	0.1
27	Simazine	Triazine	Herbicide	2.3	8.1×10^{-4}	1.62	5	201.66	-	-	-	-	-	-
28	Thifluzamide	Carboxamide	Fungicide	4.16	1.01×10^{-6}	11	7.6	528.06	-	-	-	-	-	-
29	Vinclozolin	Oxazole	Fungicide	3.02	0.02	-3.43	3.4	286.11	-	-	-	0.05	-	0.05

.

Table S2. Commodity groups and representative commodities

Commodity Group	Typical commodity categories within the group	Typical representative commodities within the category	Part of the representative commodities	Fat contents(%)
Meat	Red muscle	Beef	Sirloin	22
		Pork	Pork belly	28
	White muscle	Chicken	Drumstick	11
Fat from food of animal origin	Fat from meat	Beef	Tallow	100
		Pork	Lard	100
